# Interactions between Pea Protein Isolate and Carboxymethylcellulose in Neutral and Acid Aqueous Systems

**DOI:** 10.3390/foods10071560

**Published:** 2021-07-05

**Authors:** Ying Yue, Shujie Pang, Nana Li, Litao Tong, Lili Wang, Bei Fan, Chunhong Li, Fengzhong Wang, Liya Liu

**Affiliations:** Institute of Food Science and Technology, Chinese Academy of Agricultural Sciences/Key Laboratory of Agro-Products Processing, Ministry of Agriculture and Rural Affairs, Beijing 100193, China; nicoleyue1996@163.com (Y.Y.); asd1145068683@126.com (S.P.); lnn24680@163.com (N.L.); tonglitao@caas.cn (L.T.); wlland2013@163.com (L.W.); fanbei@caas.cn (B.F.); lichunhong18@sohu.com (C.L.)

**Keywords:** pea protein isolate, carboxymethylcellulose, electrostatic interactions, soluble complexes, stability

## Abstract

Pea protein isolate (PPI), as an emerging plant protein, has gradually aroused the attention of the public, but the PPI, especially high-concentration PPI’s low stability in the acidic aqueous system, was still a problem that limited its application. In this research, we investigated the interactions between relatively high concentrations of PPI (3.0%) and carboxymethylcellulose (CMC, 0–0.5%) in neutral and acid aqueous systems to explore the change of the phase behavior and stability of PPI as affected by CMC. It showed that the stability of PPI in the aqueous systems strongly depended on the CMC concentration, especially at the acidic aqueous systems. At neutral aqueous system, a certain amount addition of CMC into the PPI caused serious phase separation. While stable PPI solutions can be obtained at a narrow region around pH 4.5 to 5.5 by adding different amounts of CMC. The enhancement in the electrostatic repulsion and steric hindrance between the newly formed PPI-CMC biopolymers, as well as the increase in bulk viscosity with the adding of CMC at pH 4.5, contributed to the higher stability of PPI in acidic aqueous systems.

## 1. Introduction

Proteins, as their solubility, emulsifying capabilities, foaming and rheological behaviors, and some other functional properties, have been widely used in the food industry [1]. In recent years, there is a preferred alternative to replace animal-based protein sources with plant proteins due to the latter’s lower costs, sustainability, and lower carbon footprint and their benefits in cardiovascular health and physiological effects [2,3].

The pea, as the second most important leguminous crop, is rich in proteins (around 20–25%) [4]. The pea protein has been utilized as emerging plant protein ingredients in plant-based foods to replace animal-based protein due to its high nutritional value, non-GM, sustainable, and functional benefits [5]. In the food industry, plant-based beverages are often prepared with whole or peeled seeds, and protein extracts [6]. The use of pea protein isolate (PPI) in the fields of preparing protein-fortified beverages and pea milk is quite popular in recent years. However, different from other legume proteins (such as faba bean, lentil, and chickpea), PPI was reported to exhibit very low stability in the acidic aqueous system due to its poor functional characteristics [7,8,9], which limits its application in acidic protein beverages and yogurt products.

Polysaccharides have been widely used as stabilizers, thickeners, and gelling agents in varieties of products like beverages to improve their rheological characteristics and provide a desirable mouth feel [10]. Besides, the proteins could interact with oppositely charged polysaccharides to form an electrostatic complex, which increased the electrostatic repulsion and steric hindrance of the complexes, thereby improving the stability of the protein particles or the protein-stabilized emulsions [11,12,13,14]. In that case, pH plays an important role as its effects on the number of ionizable reactive groups on polysaccharides and proteins [8,12]. The mixing ratio of biopolymers is also critical for the charge balance of the systems [15].

Carboxymethylcellulose (CMC) is a typical anionic water-soluble polysaccharide (pKa = 3.5) [14]. It has been widely used in food products for its good thickening and emulsification properties and low prices [16]. The research has reported that CMC could increase the whey protein stability during aging, heating, or freezing through the complexation with whey protein [17], while the effect of the CMC on the stability of PPI in aqueous systems has not been reported. Due to high concentrations of protein-fortified beverages become more and more popular, the research of the interaction between the CMC and PPI is necessary.

This study aimed to study the associative behavior involved in the mixed PPI-CMC aqueous systems at a relatively high concentration of PPI (3.0%) at neutral and acid aqueous systems in the presence of 0–0.5% CMC. Explorations of interactions between PPI and CMC were evaluated by the phase behavior, thermodynamic changes, protein solubility, surface hydrophobicity, surface charge, and bulk viscosity. The results might provide valuable insight into the development of nutritional food fluids with high levels of PPI.

## 2. Materials and Methods

### 2.1. Materials

Raw yellow pea flour (25% protein) was provided by Jingu Grain and Oil Food Co., Ltd. (Baoding, China). CMC was kindly donated by Sinopharm (Shanghai, China). The substitution degree (DS) and the weight-average molecular weight (M_w_) of CMC were 0.92 and 360 kDa, respectively. All solutions were diluted with deionized water. All other chemical reagents were of analytical grade.

### 2.2. Extraction of PPI

PPI was prepared from yellow pea according to the method of Lan et al. [8]. After mixing the yellow pea flour and water at a ratio of 1:15 (*w*/*v*), the pH of the solution was adjusted to 9.5 with 1.0 mol/L NaOH. The mixture was stirred at 500 rpm with a magnetic stirrer at 25 °C for 1 h and centrifuged at 3400× *g* for 20 min at ambient temperature. Then precipitating proteins with 1.0 mol/L HCL by adjusting the pH of the supernatant to 4.5 and centrifuging again at 3400× *g* for 20 min to collect the pellet. The recovered pellet was washed with water and adjusted to pH 7.0 with 1.0 mol/L NaOH. Then the PPI was freeze-dried for 72 h and sealed within a 500 mL plastic screw-capped bottle at −20 °C before use. The final protein concentration of PPI power was 85.50% (N × 6.25) measured by the Kjeldahl method [18]. The molecular weight of PPI (220 kDa) was determined using gel permeation chromatography and multi-angle laser light scattering analysis (HPSEC-MALLS) [19,20]. Generally, the PPI powder was diluted to the protein concentration of 1 mg/mL with 50 mmol/L disodium hydrogen phosphate-sodium dihydrogen phosphate. As well, after the solution was filtered through a 0.45 μm filter, 200 μL was injected into a high-performance size-exclusion (HPSEC) system with a TSK-gel Super Multi pore PW-H column and eluted with 50 mmol/L disodium hydrogen phosphate-sodium dihydrogen phosphate. The flow rate was set as 0.5 mL/min and the test was performed at 25 °C. The whole running rime was 36 min.

### 2.3. Preparation of PPI and CMC Stock Solutions

The PPI power was dissolved with 10 mmol/L PBS (Phosphate Buffer solution) to prepare a 5.0% PPI solution. The pH of the PPI solution was adjusted to 8.0 using 1.0 mol/L NaOH and stirred at 500 rpm for 1 h at 25 °C to make the solution fully hydrated, then the solution was adjusted back to pH 7.0 by using 1.0 mol/L HCl and stirred for 4 h on a magnetic stirrer. The PPI solutions were stored at 4 °C overnight before use. 1.0% CMC stock solution was prepared by dissolving CMC in 0.01 mol/L PBS, stirring the solution, and heating at 45 °C for 1 h to promote hydration and stirring for 4 h at room temperature. The pH was adjusted to 7.0 and stored the CMC solution at 4 °C before use.

### 2.4. Preparation of PPI and PPI-CMC Mixtures

PPI and CMC aqueous systems containing 3.0% PPI and 0–0.5% CMC (0.1 units) were prepared by mixing different ratios of stock solutions. In summary, the PPI-CMC mixtures were prepared by adding the appropriate volume of CMC stock solutions into the PPI stock solution to achieve the required PPI-CMC concentration and stirred for at least 90 min to ensure a good dispersion. The PPI-CMC solutions were stored at 4 °C and the pH was adjusted to 7.0 before use if necessary.

### 2.5. Phase Diagram

The phase behavior of the PPI-CMC mixed aqueous system as a function of pH and CMC concentration was investigated according to the method reported by Liu et al. [14] with slight modification. Generally, the pH of each sample was gradually adjusted from 7.0 to 3.0 using 0.1 mol/L NaOH and 0.1 mol/L HCl with continuously stirring. The samples were equilibrated at 4 °C for 24 h before measurement. The visual aspect of the complexes was assessed by visual observation as a translucent solution, cloudy solution, precipitation, and cloudy solution, or precipitation and clear solution, respectively. From these observations, a phase diagram of the PPI-CMC mixed solutions at different pH and CMC concentrations was constructed.

### 2.6. Protein Solubility Measurement

The protein solubility was measured according to the previous method of Beck et al. [21] with slight modification. In brief, the PPI-CMC mixed samples (3.0% PPI, 0–0.5% CMC) were centrifuged at 11,100× *g* for 20 min at 4 °C to remove the insoluble residues. Then, the BCA kit (Beijing Solarbio Science & Technology Co., Ltd., Beijing, China) was used to determine the concentration of soluble protein in the supernatant. The protein solubility was calculated as follow:

$$\mathrm{Protein}\;\mathrm{solubility}\;(\%)=\frac{\mathrm{protein}\;\mathrm{concentration}\;\mathrm{of}\;\mathrm{supernatant}}{\mathrm{protein}\;\mathrm{concentration}\;\mathrm{of}\;\mathrm{initial}\;\mathrm{samples}}\;\times \;100\%.$$

### 2.7. Isothermal Titration Calorimetry (ITC) Test

According to the method of Wang et al. [22], the interaction energetics between PPI and CMC at pH 7.0 and 4.5 were measured using MicroCalITC200 (GE Healthcare, Milton, GA, USA). All the solutions of PPI and CMC were prepared by using 0.01 mol/L PBS and were filtered through a 0.22 μm membrane filter (Millipore Co., Milford, NH, USA) before measurement. The CMC solution was placed in a 200 μL calorimetric cell and the PPI solution was injected into a 40 μL syringe, respectively. The CMC solution (2.31 × 10^−7^ mol/L) was equilibrated in the calorimetric cell at 25 °C and titrated with 2 μL PPI (5 × 10^−5^ mol/L) for 20 times with continuously stirring the solution at 1000 rpm. Each injection lasted for 10 s with 180 s intervals between two injections. Control titrations were performed by injecting the PPI solution into the phosphate buffer without a counterpart, and the corrected raw data was obtained by subtracting the heat of dilution data of the control from the raw data. The data were acquired by the software developed by MicroCal and performed with Origin 7.0 software. The enthalpy changes (ΔH) per micromolar of PPI versus PPI/CMC molar ratio were obtained by the software. 

Thermodynamic parameters including enthalpy (ΔH) and change of entropy (TΔS) for the systems at pH 7.0 and 4.5 were calculated by iterative curve fitting of the binding isotherms. In the present state, “one independent binding site” model was used to fit the data since there weren’t two inflection points in the binding isotherm [16]. The Gibbs free energy change (ΔG) was calculated as follow:

ΔG = ΔH − TΔS

where T is the absolute temperature (298.15 K).

### 2.8. Zeta Potential Measurement

The surface charge of the individual 3.0% PPI and 0.4% CMC at pH 7.0–2.0, and their mixtures (3.0% PPI, 0–0.5% CMC, pH 7.0 and 4.5) were measured with Zetasizer Nano-ZS (Malvern Instruments Ltd., Worcestershire, UK) at 25 °C. The samples were diluted with 0.01 mol/L PBS within the same pH and ionic strength at a ratio of 1:5. The result was expressed as zeta-potential (ζ, mV) represented an average of all molecular species presented in the aqueous systems, such as free PPI and CMC molecules, or PPI/CMC complex.

### 2.9. Surface Hydrophobicity Measurement

The fluorescence spectrofluorometer (F-2500, Hitachi, Kyoto, Japan) was used to measure the protein surface hydrophobicity (PSH) as described by Reinkensmeier et al. [23] with some modifications. The PPI-CMC mixtures (3.0% PPI, 0–0.5% CMC) were diluted 150 times with 0.01 mol/L PBS (pH 4.5) and loaded into the cuvette. Then the solution was equilibrated at 25 °C and titrated with 10 successive 2 μL injections of ANS (8 × 10^−3^ mol/L) while being continuously stirred. There was 2 min between two successive injections until the final concentration of ANS was 0.16 mol/L. The constant excitation and emission slit both were 10.0 nm. The excitation wavelength was 390 nm and the emission wavelength was 470 nm. Besides, measure and subtract the absorbance of CMC solution to correct the background fluorescence. The PSH was obtained according to the initial slope of the fluorescence intensity versus protein concentration.

### 2.10. Rheological Property Test

A Rheometer Physical MCR 301 (Anton Paar, Austria), equipped with a flat plate (PP50 Ti: diameter Φ = 50 mm; 1 mm gap), was used to measure the change of the apparent viscosity of solutions with the shear rate according to Lan et al. [8]. In brief, 2.3 mL samples were loaded onto the platform of the rheometer and equilibrated for 1 min at 25 °C. Set the shear rate of 0.1 to 100 s^−1^. 

The flow behavior of the solutions was described by using the power-law model as follow [24].

(1) $$\tau =K({\dot{\gamma })}^{n}$$

where *τ* was the shear stress (Pa); *K* was the consistency index (Pa s);$\dot{\gamma }$ was the shear rate (s^−1^); *n* was the flow behavior index (dimensionless) which reflected the difference between the fluid and the Newtonian model.

### 2.11. Statistical Analysis

All measurements were performed using at least three freshly prepared samples and the data were reported as means and standard deviation. Origin 9.0 (OriginLab Corp., Northampton, MA, USA) and SPSS 22.0 (IBM SPSS statistics 24, Armonk, NY, USA) were used to analyze the data. The significant difference between the samples was analyzed using the analysis of variance (ANOVA) method at *p* < 0.05.

## 3. Results and Discussion

### 3.1. Phase Diagram

**Figure 1:**
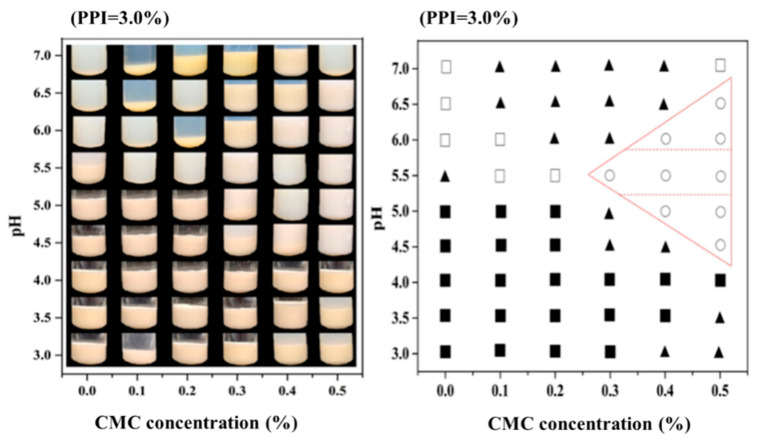
Phase diagram of pea protein isolate- carboxymethylcellulose (PPI-CMC) solutions at different pH and CMC concentrations with PPI concentration of 3.0% (□ translucent solution; ○ cloudy solution; ▲ precipitation and cloudy solution; ■ precipitation and clear solution). The labeled areas mean intermediate narrow region appeared cloudy solutions without precipitate.

Figure 1 shows the phase diagram of PPI-CMC mixed systems as affected by pH and CMC concentration. It can be seen that the individual PPI sample formed precipitate at pH ≤ 5.5, which might due to the protein syneresis and denaturation cause the aggregation of PPI. However, the mixed PPI-CMC solutions remained quite stable at a pH of 5.5. It might be the complexation of PPI and CMC provide more negative charges on the surface of the protein which inhibited the aggregation of the PPI at the acidic environment [8]. Nevertheless, it was worth noting that the addition of a certain amount of CMC led to obvious phase separation for the mixed systems prepared at neutral pHs (pH 6.0–7.0). This might due to the association of the same biopolymers inhibited the complexation of PPI and CMC, which caused the thermodynamic incompatibility between the negative charged PPI and CMC [25]. The inhibition of phase separation behavior at a higher level of polysaccharide in the neutral PPI-CMC mixed systems (pH 6.0, 0.4 and 0.5% CMC; pH 6.5 and 7.0, 0.5% CMC) could be associated with the increase in the bulk viscosity [26]. 

When the pH was further decreased to lower than 5.5, severe phase separations were observed both at: (i) 4.5 ≤ pH ≤ 5.0 and CMC concentrations < 0.4% and (ii) 3.0 ≤ pH ≤ 4.0, indicating CMC were less efficient in protecting PPI molecules during the process of acidification. The formation of these precipitates can be explained by two different mechanisms: (i) at 4.5 ≤ pH ≤ 5.0 where the pH near to protein’s pI, CMC cannot supply efficient negative charge to repel cationic amino groups on PPI surface, and extensive protein precipitates occurred via charge neutralization and bridging flocculation [27]; (ii) at pH 3.0 and 3.5, strong electrostatic interactions appeared between the two oppositely charged biopolymers, leading to forming insoluble protein/polysaccharide coacervates [28]. Besides these two distinct regions, there was a narrow region (pH 4.5, 0.5% CMC; pH 5.0, 0.4–0.5% CMC) on the phase diagram at acidic pH, where could be observed that the solutions were cloudy but precipitate was absent.

### 3.2. The Solubility of PPI–CMC Mixtures

**Figure 2:**
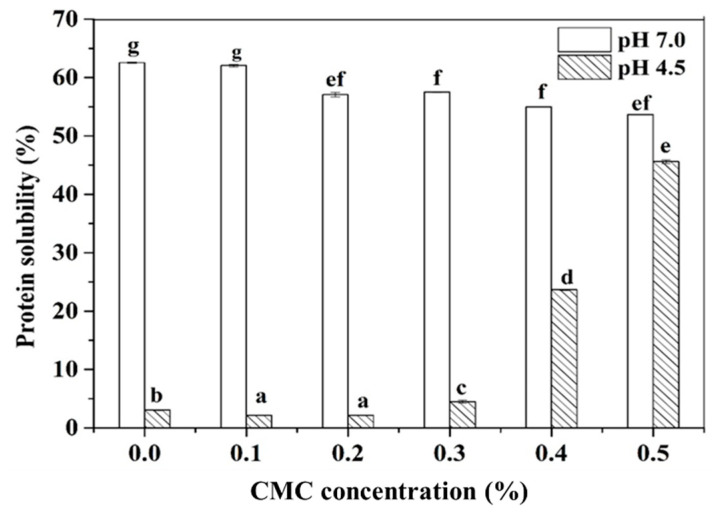
Protein solubility of 3.0% PPI solution as affected by CMC concentration at pH 7.0 and pH 4.5. Different letters represent a statistically significant difference at the same pH (*p* < 0.05).

The protein solubility of PPI-CMC solutions at different pH and CMC concentrations was showed in Figure 2. Considering the protein-fortified acid beverages or yogurt always contains pH around 4.6 [29,30], as well as the pI of the PPI around 4.5 [31], we selected pH 4.5 for further investigating the interactions between PPI and CMC and set the pH 7.0 as control. The result revealed that the protein solubility decreased gradually with the increase of CMC at pH 7.0. This result can be interpreted by the existence of thermodynamic incompatibility between PPI and CMC at higher biopolymer concentration [32], which was in agreement with the phase separation result observed in Figure 1. While the pH declined to 4.5, only about 3.0% of the PPI can be dissolved. With the addition of CMC, the protein solubility initially showed a slight decrease and then increased significantly with increasing levels of CMC.

### 3.3. Thermodynamic Characterization of Interactions between PPI and CMC

**Figure 3:**
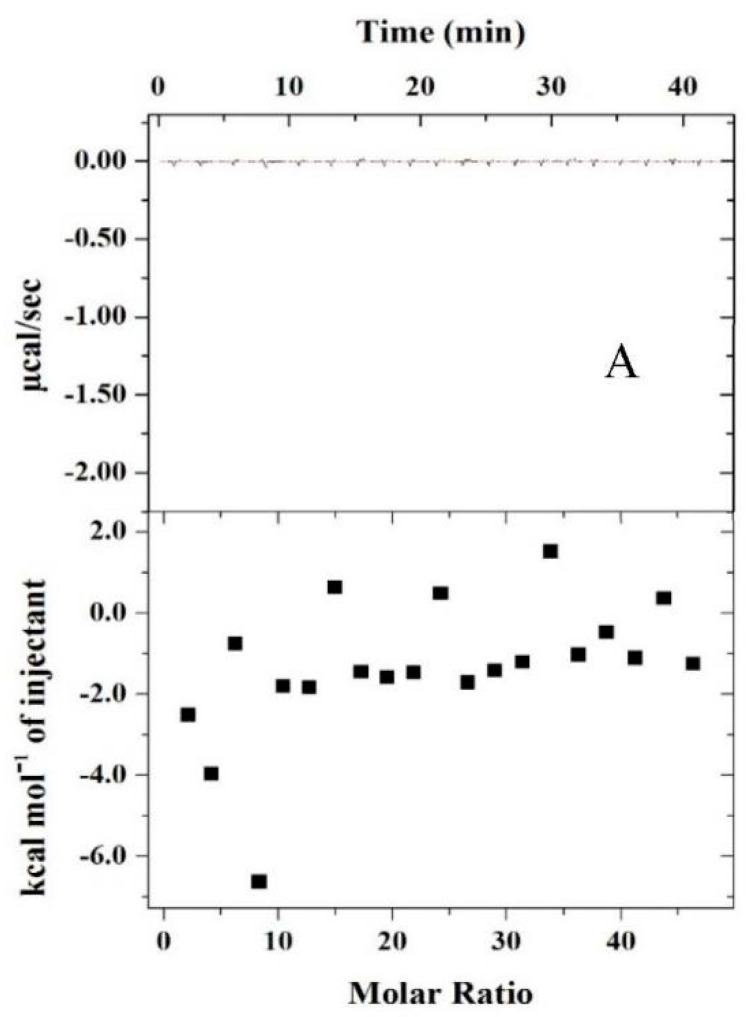

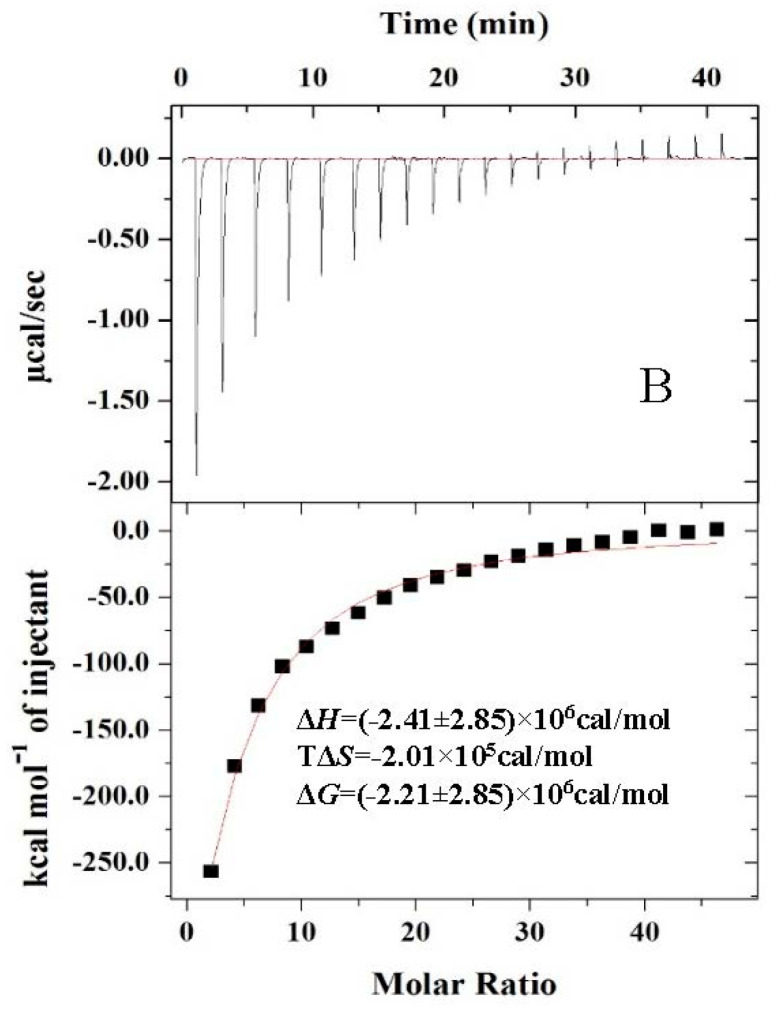
ITC titration graphs of PPI/CMC at pH 7.0 (**A**) and pH 4.5 (**B**). Upper panel: representative raw data as a function of time; lower panel: binding isotherm of enthalpy versus the molar ratio of PPI to CMC, the inserted data were the thermodynamic parameter of binding between PPI and CMC of pH 4.5 (**B**). CMC solution (2.31 × 10^−7^ mol/L) was loaded in the calorimetric cell and PPI solution (5 × 10^−5^ mol/L) was loaded in the syringe.

ITC could reflect the heat change during the interaction between molecules at a constant temperature. It could research the binding model, type, and change of energies involved in the interactions between proteins and polysaccharide have been extensively studied using this method [33,34,35]. The typical thermograms of heat rate versus time profile for PPI titrated into CMC solution at pHs 7.0 and 4.5 are presented in the top panels of Figure 3.

At pH 7.0, there was only a relatively low heat exchange during the injection process (Figure 3A). A similar phenomenon was observed in the sodium caseinate and low methoxyl pectin mixed solution prepared at pH 7.0 as reported by Wang et al. [22]. On the contrary, an obvious exothermic phenomenon was observed at 4.5, where the change of the corrected heat flow gradually decreased as the increase PPI concentration (Figure 3B). Concomitantly, the corrected heat flow at pH 4.5 reached a steady state at PPI: CMC molar ratio around 28, which implied the affinity binding sites between PPI and CMC molecules were saturated at this ratio [36]. Above this ratio, the enthalpy became positive, indicating the reaction became endothermic for hydrophobic interactions, the structure rearrangement from soluble complexes to coacervates [22,34].

Thermodynamic parameters for the system at pH 7 were not calculated by iterative curve fitting of the binding isotherms because “one independent binding site” model was not fit the complexity data of it [37]. The negative binding enthalpy changes (ΔH) indicated the PPI and CMC reactions were enthalpically favorable at pH 4.5.

The hydration effect is the main factor contributing to the entropy of complex formation [38]. It has been demonstrated that a negative TΔS can involve many contributions, and it not only due to the increased or unchanged hydration interfaces; nevertheless, a positive TΔS can be strongly indicated that water molecules are released from the complex surface [39,40]. The ΔH and TΔS values at pH 4.5 were both negative, corresponding to a general predominance of van der Waals interactions, electrostatic interactions, and hydrogen bond formation in PPI-CMC interactions. In addition, the bindings between PPI and CMC were spontaneous reactions (ΔG < 0).

### 3.4. ζ-Potential of PPI–CMC Mixtures

**Figure 4:**
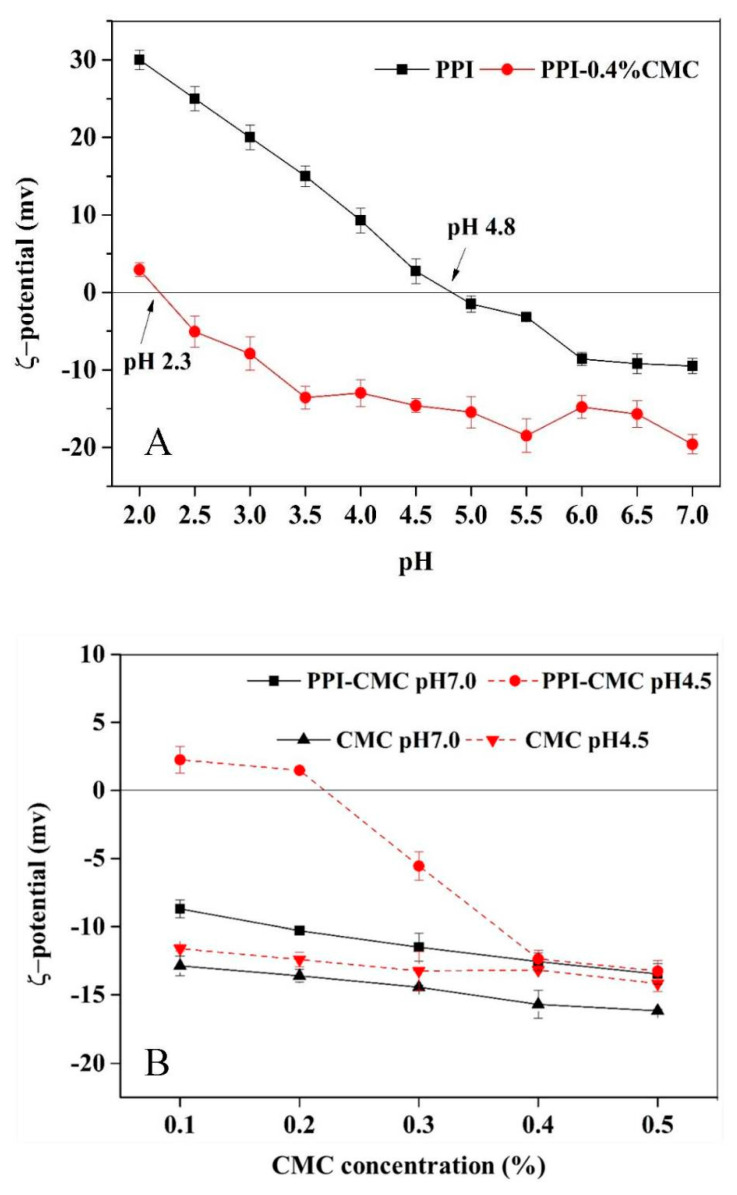
The pH-dependence of ζ-potential for PPI or CMC solutions (**A**) and the effects of CMC concentrations on the ζ-potential of 3.0% PPI solution at pH 7.0 and 4.5 (**B**).

The effects of pH on the ζ-potential of 3.0% PPI, 0.4% CMC are shown in Figure 4A. It revealed that the ζ-potential of the individual PPI solution went from negative (−9.5 mV) to positive (30 mV) as the pH decreased from 7.0 to 2.0, with a zero-charge point near pH 4.8. The CMC carried negative charges above pH 2.3 and showed no obvious change until the pH lower than 3.5.

Figure 4B shows the ζ-potential of PPI-CMC mixed solution as a function of CMC concentration at pH 7.0 and 4.5. At pH 7.0, the ζ-potential of PPI-CMC mixed samples gradually decreased with the increase of CMC, which was consistent with the trend of the individual CMC solutions. Since both PPI and CMC were negatively charged the decline of ζ-potential in the PPI-CMC mixed systems may only be attributed to the increase of the total negative charges due to the adding of anionic CMC molecules. At pH 4.5, the ζ-potential of the CMC changed unobvious with its amount, nevertheless, the ζ-potential of the PPI-CMC solutions varied from positive to negative with increasing CMC (Figure 4B). Combined with findings from ITC results, thus, it was clear that new PPI-CMC complexes were formed due to the adsorption of negative charged CMC onto the surface of positive PPI mainly through electrostatic interactions. Moreover, the ζ-potential of the PPI-CMC mixture reached a plateau value (−16.77 mV) at CMC concentration around 0.4% due to the positive charges on PPI molecules were completely saturated by CMC at its concentration close to 0.4%.

### 3.5. Surface Hydrophobicity

**Figure 5:**
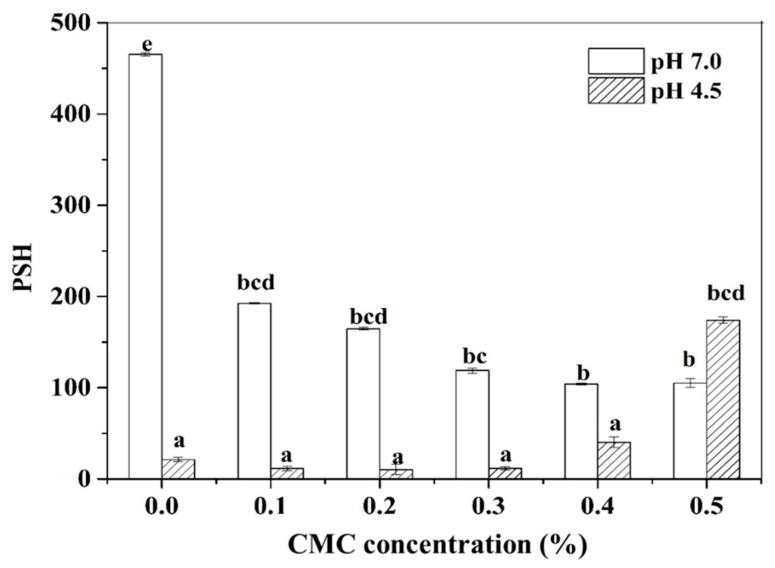
Effects of CMC concentration on the protein surface hydrophobicity (PSH) of PPI (3.0%) at pH 7.0 and pH 4.5 in the aqueous systems. Different letters indicate a statistically significant difference at the same pH (*p* < 0.05).

Surface hydrophobicity played an important role in evaluating the protein conformational changes and reflecting the number of hydrophobic groups exposed on the protein surface [41,42]. Figure 5 highlights the strong effects of CMC concentration on the PSH of PPI in the aqueous solution. It was found that the PSH decreased gradually with the addition of a higher amount of CMC at pH 7.0. This confirmed the results of ITC that hydrophobic interactions happened between PPI and CMC at pH 7.0. Therefore, the binding ability of ANS to the hydrophobic site of protein might be weakened probably due to there was an atmosphere of polysaccharides surrounded the PPI hydrophobic surface [43].

In comparison, the PSH initially showed a slight decline within the CMC concentration of ≤0.3% and then progressively increased at a CMC concentration of ≥0.4% at pH 4.5. A possible explanation to account for the initial reduction of PSH at low CMC concentration was possibly due to the enhanced hydrophobic aggregation of PPI in aqueous solutions caused by the associative effects of polysaccharide [44]. Nevertheless, higher levels of CMC produced an enhanced exposure of the protein hydrophobic segments due to electrostatic and/or steric stabilization.

### 3.6. Flow Behavior of PPI-CMC Mixtures

**Figure 6:**
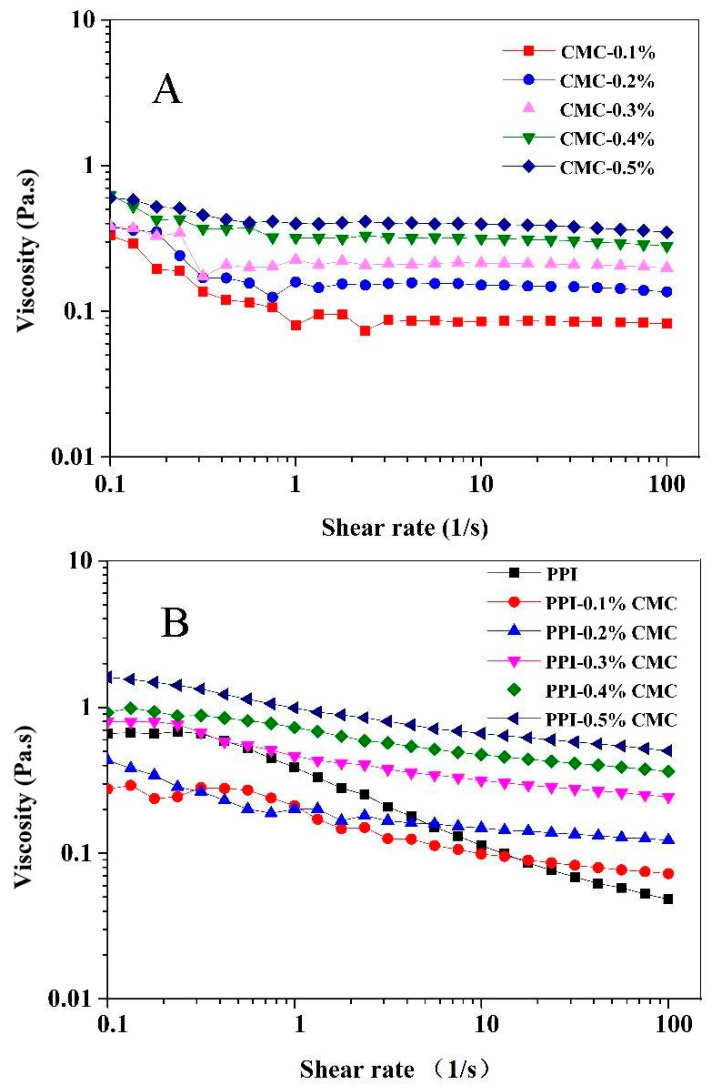

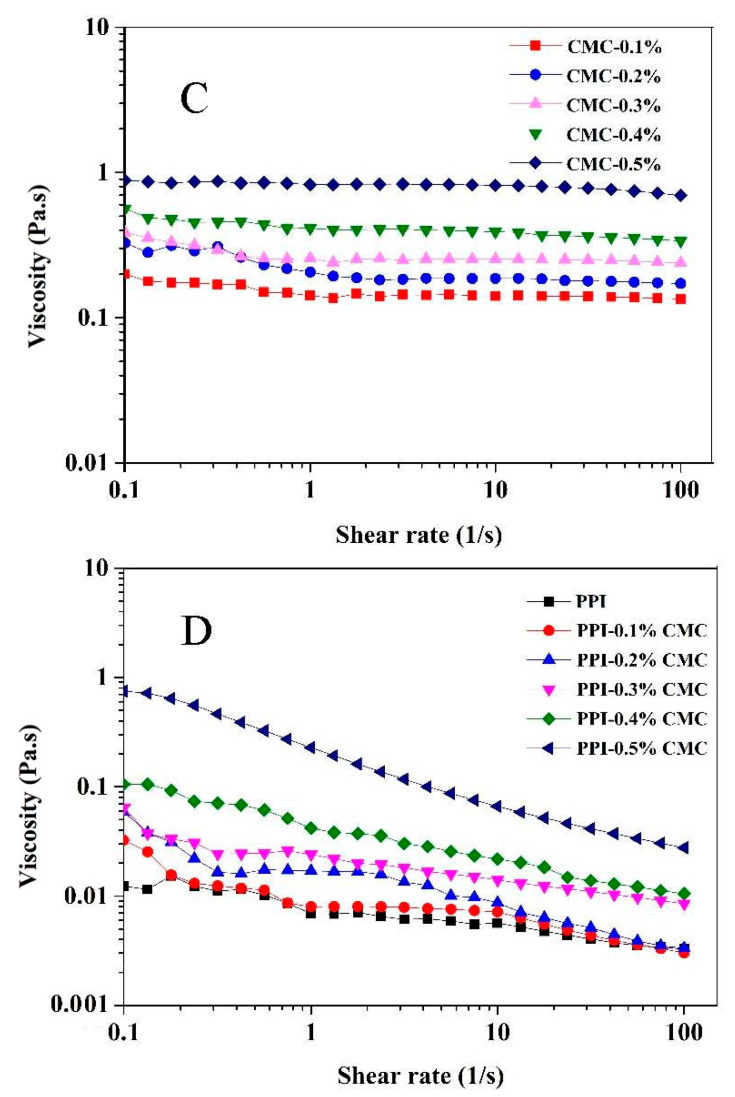
The changes of the viscosity with the shear rate of the individual CMC solution at pH 7.0 and pH 4.5 (**A**,**C**) and PPI-CMC mixed solutions at pH 7.0 and pH 4.5 (**B**,**D**).

The result of the apparent viscosity of PPI-CMC mixtures compared with the individual CMC samples is listed in Figure 6. The result indicated that the CMC solutions alone and PPI-CMC mixtures showed higher viscosity at high CMC concentration. Table 1 showed that the PPI-CMC mixed solutions prepared at pH 7.0 and 4.5 presented higher *K* values compared with the individual CMC solution at the same concentration (expect the PPI-CMC solutions with 0.5% CMC at pH 4.5). The higher *K* values for the PPI-CMC mixtures at pH 7.0 were thought to be a combined effect of protein and polysaccharides. On the other hand, considering the viscosity of PPI solution prepared at pH 4.5 was quite low (Figure 6D), the much higher *K* values of PPI-CMC mixtures compared with CMC alone at acidic pH might be the formation of new biopolymers due to the strong electrostatic attraction force and higher inter- and intramolecular interactions at acidic pH according to the ITC measurement, which could increase the resistance against flow [45].

Moreover, it was clear that most of the n values for the individual CMC solutions were close to 1 (Table 1). However, PPI-CMC mixtures exhibited n values less than 1. It is known that the flow behavior *n* = 1 represents a Newtonian fluid and *n* < 1 means a pseudoplastic (shear-thinning) fluid [46]. Therefore, the PPI-CMC mixtures showed an obvious pseudoplastic behavior with shear-thinning flow behavior, indicating the destruction of network structure between biopolymers or inter-molecular bonds, and/or reorganization of biopolymer structures under shear [45]. Meanwhile, the n values of PPI-CMC mixed solutions were much lower than that of the CMC solutions within the same pH, indicating a shift to pseudoplastic behavior and again proved the existence of PPI-CMC interactions in these aqueous systems.

## 4. Conclusions

The influences of CMC concentration and pH on the stability of PPI aqueous solutions were investigated in this research. The results showed that a certain concentration of CMC could result in the phase separation of the CMC-PPI system at the neutral pHs, while the phase separation would be inhibited when the CMC concentration increased to more than 0.4%. At the pH of 4.5, the CMC and PPI complexed through electrostatic interaction. When the CMC concentration ≥ 0.4%, the aggregate degree of PPI was reduced and the solubility of the PPI was improved. A certain concentration of CMC increased the exposure of the hydrophobic sites in PPI and improved the surface hydrophobicity of PPI. This research could provide a theoretical guideline for the application of CMC in PPI acidic beverages.

## Figures and Tables

**Table 1:** *K* and n values for CMC and PPI/CMC mixed solutions at pH 7.0 and 4.5.

	System	CMC Concentration (%)	*K* (Pa.s)	n	R
pH 7.0	CMC	0.1	0.146 ± 0.009 ^a^	0.904 ± 0.010 ^d^	0.989
		0.2	0.216 ± 0.013 ^c^	0.928 ± 0.007 ^c^	0.998
		0.3	0.218 ± 0.018 ^d^	0.977 ± 0.008 ^a^	0.999
		0.4	0.405 ± 0.008 ^e^	0.946 ± 0.018 ^b^	0.998
		0.5	0.419 ± 0.016 ^f^	0.945 ± 0.008 ^b^	0.999
	PPI/CMC	0.1	0.170 ± 0.012 ^b^	0.785 ± 0.010 ^f^	0.998
		0.2	0.217 ± 0.019 ^d^	0.846 ± 0.007 ^e^	0.992
		0.3	0.502 ± 0.028 ^g^	0.830 ± 0.007 ^e^	0.996
		0.4	0.703 ± 0.013 ^h^	0.845 ± 0.009 ^e^	0.999
		0.5	0.999 ± 0.033 ^i^	0.829 ± 0.003 ^e^	0.997
pH 4.5	CMC	0.1	0.123 ± 0.011 ^a^	0.982 ± 0.006 ^a^	0.999
		0.2	0.183 ± 0.060 ^c^	0.981 ± 0.016 ^a^	0.999
		0.3	0.223 ± 0.012 ^e^	0.996 ± 0.056 ^a^	0.957
		0.4	0.373 ± 0.014 ^g^	0.971 ± 0.015 ^a^	0.997
		0.5	0.443 ± 0.022 ^j^	0.982 ± 0.003 ^b^	0.996
	PPI/CMC	0.1	0.149 ± 0.006 ^b^	0.788 ± 0.011 ^e^	0.989
		0.2	0.208 ± 0.004 ^d^	0.854 ± 0.007 ^d^	0.978
		0.3	0.329 ± 0.003 ^f^	0.910 ± 0.007 ^c^	0.999
		0.4	0.413 ± 0.011 ^h^	0.855 ± 0.013 ^d^	0.997
		0.5	0.427 ± 0.017 ^i^	0.813 ± 0.020 ^e^	0.999

Different letters within the same column have significant differences (*p* < 0.05) at the same pH.
